# Bidirectional effects of oral anticoagulants on gut microbiota in patients with atrial fibrillation

**DOI:** 10.3389/fcimb.2023.1038472

**Published:** 2023-03-24

**Authors:** Wan Li, Changxia Li, Cheng Ren, Shiju Zhou, Huan Cheng, Yuanrong Chen, Xiaowei Han, Yiming Zhong, Licheng Zhou, Dongming Xie, Haiyue Liu, Jiahe Xie

**Affiliations:** ^1^ Department of Cardiology, Key Laboratory of Prevention and Treatment of Cardiovascular and Cerebrovascular Diseases, Ministry of Education, Jiangxi Branch, Center of National Geriatric Disease Clinical Medical Research Center, First Affiliated Hospital of Gannan Medical University, Gannan Medical University, Ganzhou, China; ^2^ Department of Emergency, The First Affiliated Hospital of Gannan Medical University, Gannan Medical University, Ganzhou, China; ^3^ Xiamen Key Laboratory of Genetic Testing, The Department of Laboratory Medicine, The First Affiliated Hospital of Xiamen University, School of Medicine, Xiamen University, Xiamen, China

**Keywords:** atrial fibrillation, gut microbiota, oral anticoagulants, inflammation, thrombosis

## Abstract

**Background:**

The imbalance of gut microbiota (GM) is associated with a higher risk of thrombosis in patients with atrial fibrillation (AF). Oral anticoagulants (OACs) have been found to significantly reduce the risk of thromboembolism and increase the risk of bleeding. However, the OAC-induced alterations in gut microbiota in patients with AF remain elusive.

**Methods:**

In this study, the microbial composition in 42 AF patients who received long-term OAC treatment (AF-OAC group), 47 AF patients who did not (AF group), and 40 volunteers with the risk of AF (control group) were analyzed by 16S rRNA gene sequencing of fecal bacterial DNA. The metagenomic functional prediction of major bacterial taxa was performed using the Phylogenetic Investigation of Communities by Reconstruction of Unobserved States (PICRUSt) software package.

**Results:**

The gut microbiota differed between the AF-OAC and AF groups. The abundance of *Bifidobacterium* and *Lactobacillus* decreased in the two disease groups at the genus level, but OACs treatment mitigated the decreasing tendency and increased beneficial bacterial genera, such as *Megamonas*. In addition, OACs reduced the abundance of pro-inflammatory taxa on the genus *Ruminococcus* but increased certain potential pathogenic taxa, such as genera *Streptococcus*, *Escherichia-Shigella*, and *Klebsiella*. The Subgroup Linear discriminant analysis effect size (LEfSe) analyses revealed that *Bacteroidetes*, *Brucella*, and *Ochrobactrum* were more abundant in the anticoagulated bleeding AF patients, *Akkermansia* and *Faecalibacterium* were more abundant in the non-anticoagulated-bleeding-AF patients. The neutrophil-to-lymphocyte ratio (NLR) was lower in the AF-OAC group compared with the AF group (*P* < 0.05). *Ruminococcus* was positively correlated with the NLR and negatively correlated with the CHA2DS2-VASc score (*P* < 0.05), and the OACs-enriched species (*Megamonas* and *Actinobacteria*) was positively correlated with the prothrombin time (PT) (*P* < 0.05). *Ruminococcus* and *Roseburia* were negatively associated with bleeding events (*P* < 0.05).

**Conclusions:**

Our study suggested that OACs might benefit AF patients by reducing the inflammatory response and modulating the composition and abundance of gut microbiota. In particular, OACs increased the abundance of some gut microbiota involved in bleeding and gastrointestinal dysfunction indicating that the exogenous supplementation with *Faecalibacterium* and *Akkermansia* might be a prophylactic strategy for AF-OAC patients to lower the risk of bleeding after anticoagulation.

## Introduction

Atrial fibrillation (AF) is the most common form of cardiac arrhythmia, characterized by irregular beating of the atria (Wang and Wang, 2020). The global prevalence of AF has been reported to be 2% to 4%, and a 2.3-fold rise is expected, owing to increasing extended life expectancy and improvement in screening tools (Hindricks et al., 2021). AF can lead to many complications, such as hemodynamic disturbances and thromboembolism, increasing the readmission rates and healthcare burden. Additionally, AF is strongly associated with thromboembolic events, with the risk of embolism being seven times higher in patients with AF than in those with sinus rhythm (Noubiap et al., 2021). The mechanisms of thrombosis in patients with AF are complex, involving multiple risk factors. Congestive heart failure, hypertension, advanced age, diabetes, previous thromboembolism, vascular disease, and being female are all associated with thrombosis (Schotten et al., 2011). The prevention of thromboembolic events is currently the main goal of AF treatment (Yalcin et al., 2015). The CHA_2_DS_2_-VASc score is a clinical prediction tool used to assess the risk of embolic complications in non-valvular AF patients, which is based on six characteristics (1 point: age (65-74 years), female, history of heart failure or left ventricular systolic dysfunction, history of hypertension, history of known atherosclerosis, and diabetes mellitus; 2 points: history of stroke or transient ischemic attack, age (more than 75 years)) (Lip et al., 2010). Oral anticoagulants (OACs) are commonly used in AF patients having CHA_2_DS_2_-VASc scores of more than 1 in men and 2 in women. The European Cardiovascular Science Guidelines for the Management of AF published in 2021 clearly stated that OACs are the cornerstone of AF management, reducing the risk of stroke and death (Chong et al., 2021).

The gut microbiota and its metabolites have been shown to play an important role in cardiovascular diseases (CVD) with the establishment of the cardiac-gut axis. Gut microbiota participates in thrombosis through inflammation reduction, endothelial cell dysfunction, platelet activation, and coagulation system activation (Formes and Reinhardt, 2019; Reinhardt, 2019; Hasan et al., 2020; Witkowski et al., 2020). A previous study showed that OACs can downregulate the expression of inflammatory factors in patients with AF (Grimnes et al., 2021). Only a few observational studies have described changes in gut microbiota in patients with AF and post-AF ablation patients. In addition, the gut microbiota was found to affect the bioavailability and efficacy of OACs (Hu et al., 2021). The above-mentioned small observational studies were not specifically designed to address the influence of drugs on the changes in gut microbiota. Therefore, we hypothesize that OACs may affect thrombosis in patients with AF by altering the intestinal microbiota. In addition, every year, 1% to 4% of AF patients receiving anticoagulation have a cardioembolic stroke or other site embolic strokes, and 2% have bleeding episodes with varying degrees of gastrointestinal side effects (Rohla et al., 2019). However, no studies focused on the effects of OACs on the gut microbiota and their relationship with the clinical phenotype of AF. Hence, this study further explored the effect of OACs on the gut microbiota of patients with AF and analyzed the correlation between microbiota and clinical phenotypes, as well as looked for biomarkers of bleeding events after anticoagulation from the perspective of the gut microbiota.

## Materials and methods

### Study design and population

We consecutively recruited 89 patients who were hospitalized in the First Affiliated Hospital of Gannan Medical University between January 1, 2021, and October 30, 2021. The inclusion criteria for patients were as follows: (1) the main admission diagnosis of patients was AF; (2) patients with AF history without radiofrequency ablation, The AF patients who had taken new OACs (NOACs) over 3 weeks, or those taking warfarin with an international normalized ratio (INR) greater than 1.5 were included in the AF-OAC group (Liu et al., 2017; Chan et al., 2019). The AF-OAC group was further subdivided into the warfarin, rivaroxaban, and dabigatran subgroups. The AF patients who had never taken any anticoagulants were in the AF group. Additionally, we also recruited 40 volunteers for the control group who met the following criteria: (1) exhibited no AF-related clinical symptoms and signs or showed negative results upon single-lead Electrocardiograph (ECG, which doctors use to see if the heart is working normally by recording the changes in electrical activity produced by the heart during each cardiac cycle.) or 24-hour Holter ECG, (2) had not taken any OACs within 3 weeks before recruitment. All subjects were included in the study only when they maintained a balanced diet at the time of enrollment and before enrollment. Subjects with one or more of the following comorbidities were excluded: (1) antibiotics, prebiotics, or probiotics used for more than 3 successive days within 3 months before admission; (2) had gastrointestinal diseases or underwent gastrointestinal surgery within a half year; (3) had any malignant tumors, autoimmune disorders, infectious diseases or serious renal or live dysfunction; (4) Total white blood cell (WBC) count was more than 10*10^12^/L or less than 4*10^12^/L.

The cohort of patients with AF who had taken OACs was followed for the event of bleeding from 1 January 2021 until 30 October 2021. The follow-up endpoint consisted of the presence of bleeding events or study endpoint.

### Genomic DNA extraction

The procedure for total fecal DNA extraction was as follows: The fecal samples of patients and control subjects were collected and the microbial genomes were extracted using the OMEGA Stool DNA Kit (OMEGA Biotech, GA, USA) following the manufacturer’s protocols. The concentration and purity of the DNA samples were determined with a Nanodrop 2000 spectrophotometer (Thermo Fisher Scientific Inc., Waltham, MA, USA).

### 16S ribosomal RNA gene sequencing

The hypervariable V3–V4 region of the bacterial small subunit (16S) rRNA gene was amplified by polymerase chain reaction (PCR) using the forward primer 341F:*5’-CCTACGGGNGGCWGCAG*-3’ and 805R:*5’-GACTACHVGGGTATCTAATCC-3’*). The amplified products were recovered and purified. The PCR products were purified using AMPure XT beads (Beckman Coulter Genomics, Danvers, MA, USA) and quantified using Qubit (Invitrogen, USA). The amplicon pools were sequenced and the sizes of the amplicon libraries were evaluated using an Agilent 2100 Bioanalyzer (Agilent, USA) and an Illumina Library Quantitation kit (Kapa Biosciences, MA, USA), respectively. All samples were sequenced on an Illumina Novaseq6000 PE250 platform (provided by Hangzhou Lianchuan Biological Information Technology Co., Ltd), as recommended by the manufacturer.

### Sequencing data analysis

Paired-end reads were assigned to samples based on their unique barcode and truncated by cutting off the barcode and primer sequence and merged using FLASH (Magoč and Salzberg, 2011)(version 1.2.8). Quality filtering of the raw reads was performed under specific filtering conditions to obtain the high-quality clean tags according to the fqtrim (version 0.94) (Supplementary data sheet **Table S1**). Chimeric sequences were filtered using the Vsearch software (version 2.3). The most recent QIIME2 (https://forum.qiime2.org) analysis was used, invoking DADA2 (Callahan et al., 2016) to denoise the data, which was comparable to clustering at 100% similarity, and then reducing redundancy to create the feature (ASV) table and feature sequence. Then according to SILVA (138.1) classifier, feature abundance was normalized using relative abundance of each sample (Supplementary data sheet **Table S2**). *Alpha* and *Beta* diversities were calculated by QIIME2, and the graphs were drawn using R software (version 3.4.4, R core team, R Foundation for Statistical Computing, Vienna, Austria). The R package “vegan” was used to calculate the Chao1 index, Shannon index, and Simpson index of each sample. Principal coordinate analysis (PCoA) was performed using the R program “ade4” according to the relative abundance of microbial species and the Adonis test was applied to test for significant differences between groups to assess the diversity between samples. Blast was used for sequence alignment, and the feature sequences were annotated with the SILVA database (138.1) for each representative sequence. A rarefaction curve obtained from the number of sequences extracted and the Venn diagram created from the ASVs in each group were generated using R software (version 3.4.4). The classified composition of each group was visualized as a stacked bar plot at the phylum level and as a chord plot at the genus level using R software (version 3.4.4). The Kruskal-Wallis H-test was used to compare bacterial abundance (*P* < 0.05, *q* < 0.1). The Mann–Whitney *U* test was used to compare genus bacterial abundance between AF-OAC and AF groups (*P* < 0.05). Heat maps were constructed based on the abundance at the genus and species level using the R software (version 3.4.4). The linear discriminant analysis (LDA) effect size (LEfSe) analysis was used to evaluate the differentially abundant taxon (Segata et al., 2011). *P* value were corrected for multiple testing with the Benjamini and Hochberg method to control false discovery rate (FDR). The functional profiles of microbial communities were predicted using PICRUSt2 based on Kyoto Encyclopedia of Genes and Genomes (KEGG) database (Douglas et al., 2020). Graphical representations of the results were created using statistical analysis of metagenomic profiles(Parks et al., 2014) (STAMP) and the calculation of *P* values was performed with Welch’s t-tests. The R (version 3.4.4) function “cor.test” with the Spearman method was used to evaluate the correlations between differential microbiota and clinical indicators.

### Statistical analysis

All clinical data were statistically analyzed using the SPSS software version 26.0 (IBM Corporation, NY, USA). Continuous variables with normal distribution were presented as mean ± standard deviation. Continuous variables with nonnormal distribution were presented as median with interquartile range (IQR). One-way analysis of variance was employed in cases of continuous variables with normal distribution for comparing the differences in clinical characteristics among the three groups. The LSD-t test was used for *post hoc* comparisons in cases of equal variance, and Kruskal-Wallis H-test was applied in cases of unequal variance. The Kruskal-Wallis H-test was also applied for continuous data that were not normally distributed among three groups, and the Mann–Whitney *U* test was for comparisons between two groups. Qualitative data were presented as a percentage, and the *χ* ^2^ or Fisher’s exact test was used for between-group comparisons. To verify the prognostic value of differential microbiota for bleeding patients after using OAC, receiver operator characteristic (ROC) curves were generated by plotting Sensitivity% versus (100%-specificity%) and the areas under the curves (AUCs) were calculated using SPSS software. All statistical tests were 2-sided, and *P < 0.05* was considered significant.

## Results

### Characteristics of the study population and clinical parameters

We investigated the gut microbiota associated with OACs by analyzing 133 fecal microbial DNA samples through 16S rRNA gene sequencing. The samples from four participants were removed from the whole feature sequence analysis due to a low number of sequence reads, and ultimately 129 participants were enrolled in this study. All participants were further divided into the control group (N = 40), AF group (N = 47), and AF-OAC group (N = 42) according to diagnosis and treatment received. The baseline characteristics of the three groups were shown in **Table 1**. Significant differences were found in the serum creatinine (Scr) level, estimated glomerular filtration rate (eGFR), total cholesterol (TC) level, and low-density lipoprotein (LDL) level between the three groups (*P < 0.05*). Only the Scr level and eGFR were significantly different between the AF and AF-OAC groups (*P* < 0.01). The Scr level was higher and eGFR was lower in the AF-OAC group, indicating that OAC impaired renal function and further increased the risk of bleeding. We assumed that the difference in disease severity between the AF-OAC and AF groups was inconspicuous at baseline according to the historical records and laboratory data.

**Table 1:** Baseline characteristics of the study cohort[ x̅ ± S, N(%)].

	AF-OAC (N = 42)	AF (N = 47)	Control (N = 40)	*P*-value
Age (year)	70.07 ± 12.22	64.36 ± 13.45	64.22 ± 12.21	0.060
Subgroup of age				
30-49	2 (4.80)	7 (14.90)	6 (15.00)	0.290
50-64	10 (23.80)	15 (31.90)	13 (32.50)	
≥65	30 (71.40)	25 (53.20)	21 (52.50)	
Female	21 (50.0%)	19 (40.4%)	24 (60.0%)	0.190
BMI (kg/m^2^)	22.69 ± 2.86	22.78 ± 3.23	23.67 ± 3.13	0.280
Type of AF				
PAF	6 (14.3%)	14 (29.8%)	NA	0.080
PsAF	36 (85.7%)	33 (70.2%)	NA	
Risk factors				
HTN	22 (52.4%)	22 (46.8%)	17 (42.5%)	0.660
T2DM	8 (19.0%)	5 (10.6%)	8 (20.0%)	0.420
CHD	11 (23.8%)	8 (17.0%)	12 (30.0%)	0.359
HF	9 (21.4%)	8 (17.0%)	NA	
Medications				
Stain	17 (40.5%)	13 (27.7%)	12 (30.0%)	0.400
CCB	14 (33.3%)	10 (21.3%)	15 (37.5%)	0.225
ACEI	2 (4.7%)	3 (6.4%)	4 (10.0%)	0.215
ARB	8 (19.0%)	5 (10.6%)	2 (5.0%)	0.050
Asprin	13 (31.0%)	9 (19.1%)	8 (20.0%)	0.354
Clopidogrel	11 (26.2%)	6 (12.8%)	10 (25.0%)	0.224
Laboratory data				
TC (mmol/L)	3.55 ± 0.97	3.85 ± 1.12	4.34 ± 1.12	0.004
LDL (mmol/L)	2.16 ± 0.95	2.28 ± 0.77	2.66 ± 0.97	0.034
AST (U/L)	24.26 ± 10.83	23.04 ± 11.28	22.68 ± 14.91	0.804
ALT (U/L)	21.29 ± 16.38	19.09 ± 14.86	20.20 ± 13.82	0.790
Scr (mg/dL)	97.52 ± 26.19	82.26 ± 21.34	75.13 ± 18.49	<0.01^a^
eGFR (ml/min/1.73m2)	79.88 ± 29.06	103.83 ± 38.10	109.05 ± 28.76	<0.01^a^
CHA2DS2-VASc score	3.31 ± 1.56	2.04 ± 1.39	NA	<0.01

AF, Atrial fibrillation; OAC, oral anticoagulants. Hypertension (HTN) was defined as blood pressure of more than 140/90 mmHg or the use of antihypertensive drugs. Type 2 diabetes mellitus (T2DM) was defined as HbA1c more than 6.5% (NGSP), use of oral anti-diabetic drugs, or insulin therapy. Calcium channel blockers. ACEI, Angiotensin-converting enzyme inhibitor; ALT, alanine aminotransferase; ARB, angiotensin receptor blocker; AST, aspartate aminotransferase; BMI, body mass index; TC, total cholesterol; Scr, serum creatinine; LDL, low-density lipoprotein; eGFR, estimated glomerular filtration rate [it was estimated by Modification of Diet in Renal Disease (MDRD) formula]. NGSP, National Glycohemoglobin Standardization Program; NA, not available. a: AF vs AF-OAC; b: AF vs control.

The neutrophil-to-lymphocyte ratio (NLR) was significantly higher in the AF group compared with the control group, and it was much higher in the AF group compared with the AF-OAC group (*P* < 0.05). Besides, the participants in the AF-OAC group had larger left anterior diameters (LADs) and lower left ventricular ejection fraction (LVEF) (*P* < 0.05) (**Table 2**). Different OACs had different effects on coagulation; warfarin mainly prolonged prothrombin time (PT) and activated partial thromboplastin time (APTT), dabigatran prolonged thrombin time (TT), and rivaroxaban prolonged APTT (**Table 3**). In the AF-OAC group, the median follow-up time was 4.87 months (IQR: 4.0-7.5 months) and bleeding events were observed in 12 participants (warfarin subgroup: 6 patients; rivaroxaban subgroup: 5 patients; and dabigatran subgroup: 1 patient).

**Table 2:** Comparison of the inflammatory index and echocardiographic differences among the three groups ( x̅ ± S).

	AF-OAC (N = 42)	AF (N = 47)	Control (N = 40)	*P-*value
LADs(mm)	50.81 ± 9.62	45.67 ± 7.58	37.85 ± 3.51	<0.001^ab^
LVEDd(mm)	48.62 ± 6.15	48.27 ± 5.52	45.80 ± 4.32	0.039^b^
LVEF(%)	56.61 ± 8.49	60.40 ± 8.57	61.54 ± 5.39	0.011^a^
NLR	2.11 ± 0.97	2.78 ± 1.60	2.05 ± 0.75	0.006^ab^

LADs, Left atrial diameters; LVEDd, left ventricular end-diastolic dimension; LVEF, left ventricular ejection fraction; NLR, neutrophil-to-lymphocyte ratio. a: AF vs AF-OAC; b: AF vs control.

**Table 3:** The effect of different types of anticoagulants on coagulation [M(P25, P75)].

	Warfarin (N = 15)	Rivaroxaban (N = 18)	Dabigatran (N = 9)	*P-*value
APTT(s)	37.40 (31.90,41.40)	26.70 (25.28,31.15)	31.20 (26.15,43.10)	0.001^ce^
TT(s)	16.40 (16.10,17.40)	16.05 (15.45,16.60)	20.55 (15.96,54.83)	0.017^e^
PT(s)	23.40 (20.00,35.50)	12.65 (11.38,12.90)	14.05 (12.43,16.30)	<0.001^cd^

APTT, Activated partial thromboplastin time; PT, prothrombin time; TT, thrombin time. c: warfarin subgroup vs rivaroxaban subgroup; d: warfarin subgroup vs dabigatran subgroup; e: rivaroxaban subgroup vs dabigatran subgroup.

### Overview of the gut microbiome in the different group

**Figure 1:**
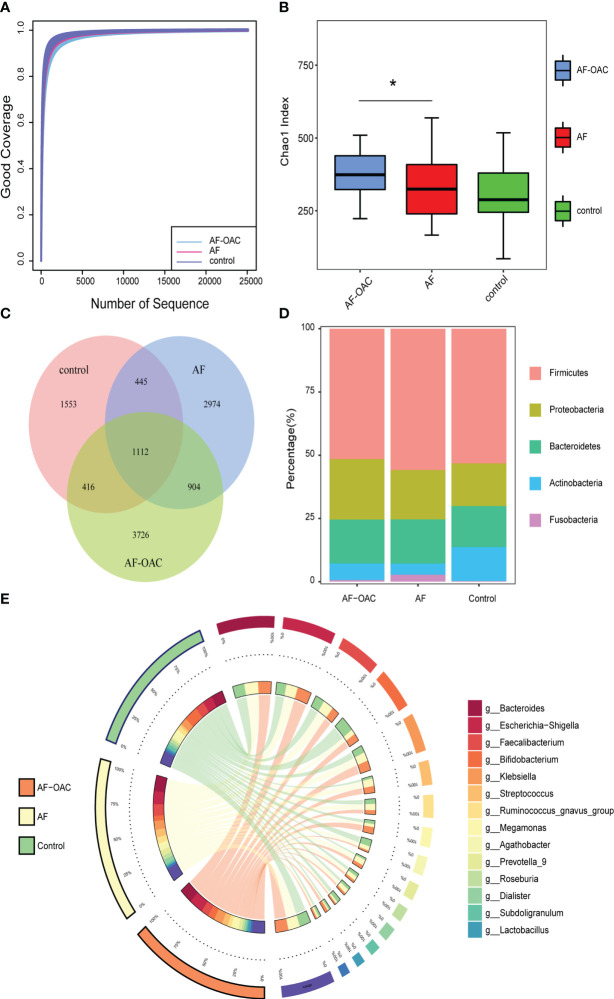
Overview of the gut microbiome in different groups. **(A)** Rarefaction analysis showed that the estimated ASV richness approached saturation in each group. **(B)** Alpha diversity, estimated by the Chao1 index, increased in the AF-OAC group, ^*^P < 0.05. **(C)** Venn diagram showing the existence of ASVs in each group. **(D)** Dominant phyla in each group. **(E)** Dominant genera and their contribution to each group.

In the present microbiome study, 8,102,793 high-quality 16S rRNA reads were obtained, with a median read count of 53,228 (range: 25,093 to 68,706) per sample. The total number of features was 11,728 after denoising (Supplementary data sheet **Table S2**). The rarefaction curves of richness (sob index) indicated the adequacy of the sequencing depth (**Figure 1A**). Regarding the alpha diversity, we observed significant differences only in the Chao1 index between the AF and AF-OAC groups (*p <0.05*) (**Figure 1B**). Shannon and Simpson indexes showed no significant differences between the three groups (**Supplementary Figures S1A–B**). We evaluated the beta diversity based on the weighted UniFrac PCoA plots to assess the overall structure of the gut microbiota. The PCoA plots (**Supplementary Figure S1C**) revealed no separation of the AF group and the control group or AF-OAC group (AF-OAC versus AF: R^2^ = 0.02, *P* = 0.19, AF versus control: R^2^ = 0.02, *P*= 0.07, Adonis test). Additionally, no significant differences were found in α and β diversities in the three anticoagulated subgroups (**Supplementary Figure S2**). Our results showed that the overall gut microbiota composition of participants in the AF-OAC group exhibited no significant difference compared with that in the AF group.

A Venn diagram was constructed to examine the existence of ASVs with a relative abundance of more than 0.1% in each group (**Figure 1C**). 1,112 ASVs were shared by all three groups. However, only a total of 904 ASVs were specifically shared between the two disease groups. In addition, the AF, AF-OAC, and control groups had 2,974, 3,726, and 1,553 ASVs, respectively.

The relative proportion of dominant taxa at the phyla level was assessed and five phyla were identified in each group (**Figure 1D**). *Firmicutes* was the most dominant phylum, with a relative proportion of 50.8%, 55.07%, and 52.40% in the AF-OAC, AF, and control groups, respectively. The second most dominant phylum was *Proteobacteria*, (AF-OAC group: 23.60%, AF group:19.25%, control group:16.57%). Other observed phyla included *Bacteroidota*, *Fusobacteria*, and *Actinobacteria*. The abundance of *Fusobacteria* and *Actinobacteria* was significantly different between the AF and control groups (*P* < 0.05, *q<0.05*). In addition, the abundance of *Actinobacteria* was significantly different between the AF-OAC and AF groups (*P* < 0.05, *q*<0.05) (**Table 4**). Otherwise, we also found that the dominant taxa in the anticoagulated subgroups were consistent with those in the AF-OAC group at the phylum level (**Supplementary Figure S3**).

**Table 4:** Comparison of differential phylum abundance in TOP5 among AF-OAC, AF and control groups [M(P25, P75)].

	AF-OAC (N = 42)	AF (N = 47)	Control (N = 40)	*P-*value	*q-*value
*Actinobacteria*	0.1 (0.07,0.25)	0.06 (0.01,0.18)	0.01 (0.01,0.15)	<0.05^ab^	<0.05^ab^
*Fusobacteria*	0.11 (0.05,0.39)	0.14 (0.08,1.21)	0.07 (0.01,0.18)	<0.05^b^	<0.05^b^

a: AF vs AF-OAC; b: AF vs control.

The top 15 most abundant genera at the genus level and their contribution to each group are shown in **Figure 1E**. *Bacteroides*, accounting for 25.80% of all samples, was the most predominant genus. Although the difference was not statistically significant, *bacteroides* tended to be more abundant in the AF group than in the AF-OAC group (AF-OAC: 17.0%, AF: 18.3%, *P* = 0.089), which aligned well with a previous report(Zuo et al., 2019). *bacteroides* that produce penta- and tetra-acylated lipid A has been reported to reduce colonic inflammation, endotoxemia, and atherosclerosis in *ApoE^−/−^* mice (Wrzosek et al., 2013).

### Alterations in the composition of the microbiota profile associated with OAC Use

**Figure 2:**
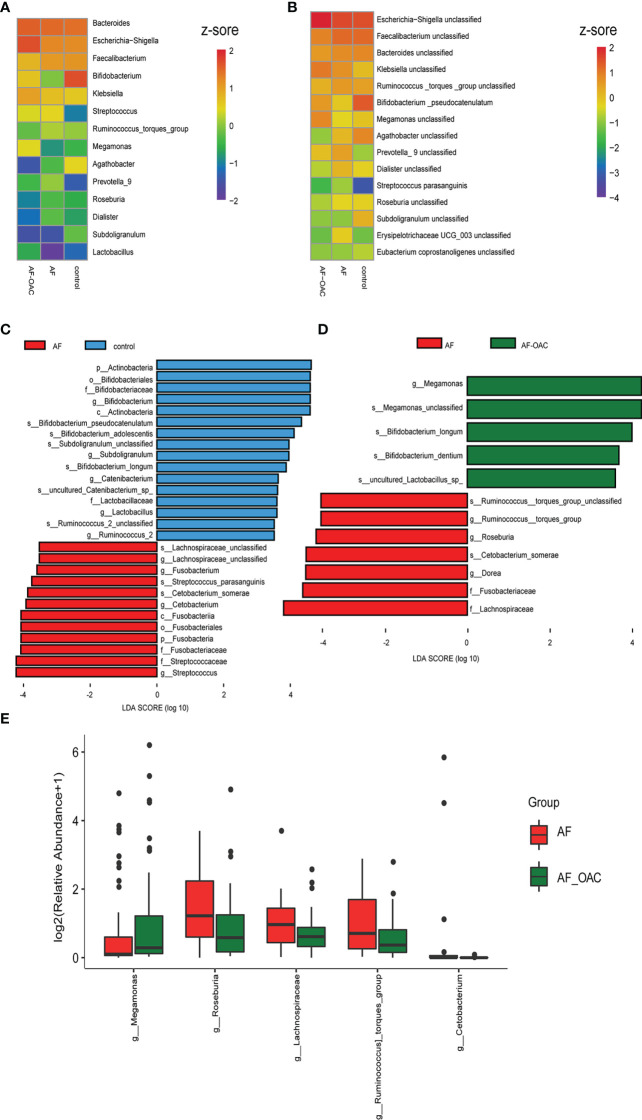
Common taxa and biomarkers in the three groups. **(A)** Heat map of the top 15 shared genera. Abundance profiles transformed Z-scores *via* average abundance subtraction and division by the standard deviation. Negative (blue) and positive (red) Z-scores revealed that the row abundance levels were lower and higher compared with the mean. **(B)** Heat map of the first top 15 shared species. The abundance profiles were analyzed as in A. **(C)** Taxonomic cladogram obtained using linear discriminant analysis (LDA) effect size (LEfSe) analysis and Wilcoxon test of the 16SrRNA gene sequences. LEfSe analysis identified the taxa with the largest differences in abundance between the AF and control groups. **(D)** Largest difference in taxa between the AF and AF-OAC groups obtained by LEfSe analysis. **(E)** Differences in the relative abundance of intestine microbiota between AF and AF-OAC groups at the genus level (Mann–Whitney *U* test).

We assessed the taxa that were changed in the gut of AF and AF-OAC patients at both the genus and species levels. The relative abundance of genera *Bifidobacterium* and *Lactobacillus* decreased in the AF-OAC and AF groups compared with the control group. However, we identified a dramatic decline in them in the AF group. In addition, the abundance of other genera, such as *Ruminococcus*, *Streptococcus*, and *Dialister*, as well as that of species including *Ruminococcus torques group unclassified*, *Streptococcus parasanguinis*, and *Dialister* unclassified, exhibited a progressively increasing trend in patients with AF. The genera *Escherichia-Shigella* and *Klebsiella* were more abundant in the AF-OAC group (**Figures 2A, B**).

Furthermore, LEfSe analysis was used to identify the specific bacteria associated with OACs and AF. Between the AF and control groups (**Figure 2C**, Supplementary data sheet **Table S3**), the abundance of the members of the phylum *Actinobacteria*, genera *Bifidobacterium*, *Subdoligranulum* and *Lactobacillus* were significantly higher in the control group (LDA scores (log10) >3.5, *P < 0.05*, FDR corrected). The relative abundance of the members of the family *Lachnospiraceae*, genus *Streptococcus*, and *Fusobacterium* were enriched in the AF group (LDA scores (log10) >3.5, *P < 0.05*, FDR corrected). Otherwise, LEfSe analysis revealed 12 discriminative features (LDA scores (log10) > 3.5, *P < 0.05*, FDR corrected) at the family (n = 2), genus (n = 4) and species (n = 6) levels between the AF and AF-OAC groups (**Figure 2D**, Supplementary data sheet **Table S4**). Genera *Bifidobacterium*, *Lactobacillus* (which are inhabitants of the gastrointestinal tract and commonly considered a group of probiotics(Wu et al., 2016)), and *Megamonas*, and phylum *Actinobacteria* were overexpressed in the AF-OAC group than in the AF group, while the abundance of the following five species were increased in the AF group: the genera *Roseburia*, *Dorea*, and *Streptococcus*, family *Lachnospiraceae*, and the species *Ruminococcus torques group*. *Roseburia* is an anaerobic bacterium that ferments intestinal carbohydrates to promote the production of short-chain fatty acids (SCFAs), and SCFAs have been shown to have a protective effect on the cardiovascular system (Zhernakova et al., 2016; Jie et al., 2017).

The relative abundance of the genera *Megamonas* was significantly higher in the AF-OAC group (*P* < 0.05, FDR corrected), whereas the genera *Roseburia, Ruminococcus torques group*, and *Lachnospiraceae* were reduced by OACs treatment (**Figure 2E**). Some of the results overlap with those of LEfSe analysis, suggesting that these microbes might be the target microbiota of OACs. Thus, the balanced state in the gut was likely disrupted and OACs increased the level of probiotics in patients with AF to some degree, while reducing the production of SCFAs and promoting the production of some potentially pathogenic bacteria.

**Figure 3:**
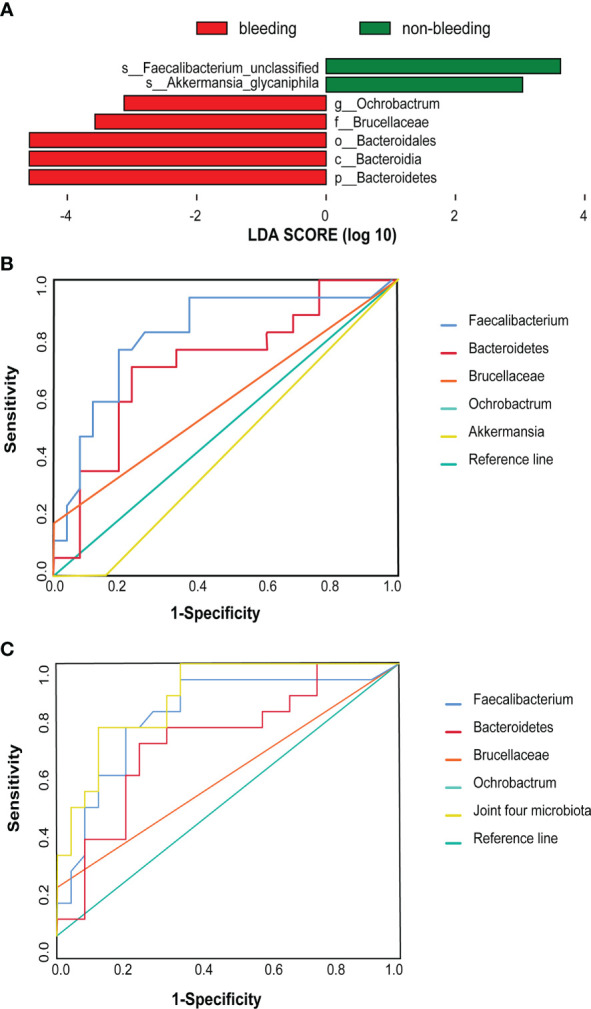
Difference taxa between the bleeding and non-bleeding subgroups. **(A)** LEfSe analysis of gut microbiota between the two subgroups. **(B)** These five taxa were selected based on LEfSe analysis and the best predictive taxa with the highest AUC [*Bacteroidetes* (AUC = 0.72, 95% CI (0.56–0.88), *P* < 0.05] and *Faecalibacterium* [AUC 0.76, 95% CI (0.49–0.85), *P* < 0.05)). **(C)** Joint ROC cure [AUC = 0.88, 95% CI (0.78–0.98), *P < 0.05*].

In addition, LEfSe analysis was used to find biomarkers of bleeding events in the AF-OAC group. Several microbiotas, including *Bacteroidetes*, *Brucella*, and *Ochrobactrum* were all significantly overrepresented (all LDA scores(log10) > 3.5, *P < 0.05*) in the AF-OAC bleeding subgroup, whereas *Akkermansia* and *Faecalibacterium* were the most abundant microbiota in the non-bleeding subgroup (LDA scores (log10)> 3.5, *P < 0.05*) (**Figure 3A**). Five species were further selected as potential bacterial markers based on the area under curve (AUC) values. Ultimately, *Bacteroidetes* (AUC = 0.72, 95% CI (0.56-0.88), *P < 0.05*) and *Faecalibacterium* (AUC = 0.76, 95% CI (0.49-0.85), *P < 0.05*) were identified as the potential bacterial marker to discriminate the bleeding risk in patients with AF (**Figure 3B**). Otherwise, we plotted the receiver operating characteristic curve for the four bacteria (*Faecalibacterium*, *Bacteroidetes*, *Brucella*, and *Ochrobactrum*) jointly (AUC = 0.88, 95% CI [0.78-0.98], *P< 0.05*), and the AUC was larger than that of the aforementioned two microbiota (**Figure 3C**).

### Relationship between the clinical indexes and gut microbiota associated with OAC therapy in AF patients and the metagenomic functional prediction

**Figure 4:**
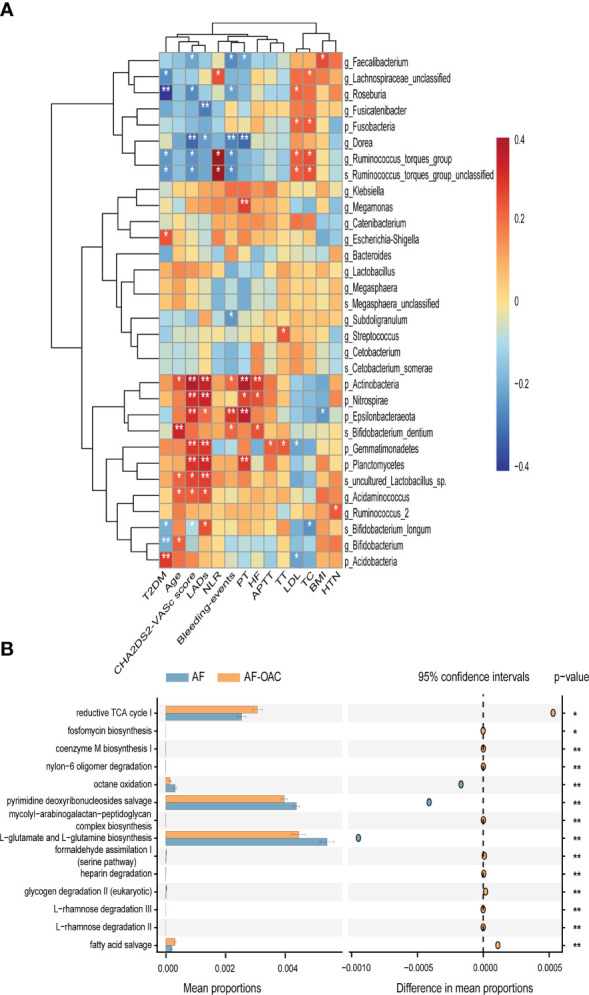
Relationship between gut microbiota and clinical indexes, the different pathways of the two disease groups predicted by PICRUSt2. **(A)** Heat map of Spearman’s rank correlation coefficient among clinical indexes and gut microbiota. Red cells indicate positive correlations, whereas blue cells indicate negative correlations. **(B)** STAMP plot shows the differential pathways between the AF and AF-OAC groups. ^*^P < 0.05, ^**^P < 0.01.

We also performed a correlation analysis of the OAC-associated gut microbiota and clinical phenotypes using the Spearman correlation method (**Figure 4A**). Notably, the abundance of OAC-enriched species (genus *Megamonas* and phylum *Actinobacteria*) were positively related to the PT (genus *Megamonas*: *r = 0.274*, *P = 0.009*; phylum *Actinobacteria*: *r = 0.388*, *P = 0.000*). Genus *Ruminococcus torques group* and family *Lachnospiraceae* were relatively depleted in AF-OAC group, and their abundance showed positive correlations with NLR (species *Ruminococcus torques group*: *r = 0.232*, *P = 0.028*; family *Lachnospiraceae*: *r = 0.223*, *P = 0.030*). Genus *Ruminococcus torques group* also showed positive correlations with the CHA_2_DS_2_-VASc score (*r = 0.244*, *P = 0.020*) and LDL level (*r = 0.225*, *P = 0.033*). Species *Bifidobacterium longum* and genus *Roseburia* showed inverse correlations with the CHA_2_DS_2_-VASc score (genus *Roseburia*: *r = -0.25*, *P = 0.015*, species *Bifidobacterium longum*: *r = -0.230*, *P = 0.020*). Species *bifidobacterium longum* was associated with TC (*r = -0.241*, *P = 0.020*). Species *uncultured_Lactobacillus_sp* was negatively correlated with the CHA_2_DS_2_-VASc score (*r = 0.267*, *P = 0.010*). Genera *Ruminococcus torques group* (*r = –0.253, P = 0.012*), *Faecalibaterium (r = –0.269, P = 0.014)* and *Roseburia* (*r = –0.216*, *P = 0.040*) were negatively correlated with bleeding events, but species *Bifidobacterium dentum* (*r = 0.218*, *P = 0.04*) had a positive correlation.

The metagenomic pathways were predicted using the PICRUSt2 tool. Five differentially abundant pathways were detected between the AF and AF-OAC group (*P < 0.05*), and four pathways (octane oxidation, reductive TCA cycle I, L-glutamate and L-glutamine biosynthesis, pyrimidine deoxyribonucleosides salvage) were downregulated, and one pathway (fatty acid salvage) was upregulated in the AF-OAC group (**Figure 4B**).

## Discussion

AF is the most prevalent sustained cardiac arrhythmia, and its pathogenesis is complex, with recent studies suggesting that inflammation mediates the development of AF (Li and Brundel, 2020). An earlier meta-analysis showed that the NLR is an independent predictor of AF (Shao et al., 2015). The NLR was found to be associated with the presence of left atrial thrombus in patients with AF (Saliba et al., 2015). Our study showed that the NLR was significantly higher in the AF group than that in the control group, while the NLR was significantly lower in the AF-OAC group than in the AF group, suggesting that OACs might decrease the risk of thrombosis by reducing inflammation, which is consistent with findings of previous study (Grimnes et al., 2021). Numerous lines of evidence linked various facets of inflammation to heighten the risks of thrombosis. The role of inflammatory pathways in thrombosis has recently been confirmed by the CANTOS clinical trial (Crossman and Rothman, 2018).

To date, only a few observational studies have described changes in gut microbiota in patients with AF. (Zuo et al., 2019; Zuo et al., 2020). Our study also found AF patients had gut dysbiosis which was consistent with the previous study. Phylum *Actinobacteriota*, order *Bifidobacteriales*, family *Bifidobacteriaceae*, genus *Bifidobacterium*, and species *Bifidobacterium longum* and phylum *Firmicutes*, order *Lactobacillales*, famiy *Lactobacillaceae*, genus *Lactobacillus*, which inhabited the gastrointestinal tract and are commonly considered a group of probiotics sharply decreased in the AF patients. Additionally, some potential pathogens (family *Lachnospiraceae*, genra *Streptococcus and Ruminococcus torques group*) increased in AF patients. OACs treatment mitigated the decreasing tendency of *Bifidobacterium* and *Lactobacillus* in patients with AF. *Bifidobacterium longum* is one of the main species of commensal bacteria present in the human digestive tract, and some strains are considered probiotic microorganisms (Hidalgo-Cantabrana et al., 2017). The species *Bifidobacterium longum*, which was found to be OACs-positive in our study, has been reported to be a potential supplement in anti-obesity therapy (Odamaki et al., 2018). Oral administration of species *Bifidobacterium longum* reduced the serum lipid levels in mice fed with a high-fat diet (Wu et al., 2020). In this study, the species *Bifidobacterium longum* was found to be negatively correlated with TC. However, we also observed that some taxa of genera *Bifidobacterium* and *Lactobacillus unclassified* were OACs-positive and associated with unfavorable clinical phenotypes, suggesting that *Bifidobacterium* and *Lactobacillus* functioned in a species/OACs-dependent manner. This result was likely attributed to the controversial role of taxa.

The Firmicutes phyla, species *Ruminococcus torques* group, is a bacterium that has previously been associated with inflammatory bowel diseases and low gut microbial richness (Jie et al., 2017). Previous research reported that high level of genus *Ruminococcus torques group* in atherosclerotic CVD, which facilitated disease progression. *Ruminococcus* can promote the generation of trimethylamine-N-oxide (TMAO) which is mainly derived from red meat, egg yolks, dairy products, and seafood. (Zhu et al., 2016). TMAO was found to enhance platelet activity, induce the expression of endothelial tissue factor, and activate exogenous coagulation pathways, which in turn caused thrombosis in patients with AF (Zhu et al., 2016; Hasan et al., 2020). In addition, TMAO suppressed the bile acid (BA) pool size and, thereby cholesterol clearance in the host (Koeth et al., 2013). BAs are essential for increasing energy expenditure and insulin sensitivity as well as reducing inflammation, which may play an important role in AF. However, *Ruminococcus torques group* has also been shown to be able to utilize mucin in the human intestine, sustaining the integrity of the intestinal mucosal barrier (Kaczmarczyk et al., 2021). Our study also showed that the abundance of genus *Ruminococcus torques* group and family *Lachnospiraceae* were relatively lower in the AF-OAC group, these taxa were positively correlated with the NLR and TC. A high abundance of *Lachnospiraceae* impaired glucose and lipid metabolism, leading to inflammation and promoting the onset of metabolic disorders (Vacca et al., 2020), or causing inflammatory conditions such as inflammatory bowel disease (IBD) or chronic kidney disease (CKD) involving the *Lachnospiraceae* family or specific taxa of *Lachnospiraceae*. Otherwise, LEfse analysis revealed that the abundance of the genus *Megamonas* was increased in AF-OAC patients. These bacteria are known to be beneficial through their involvement in the fermentation of carbohydrates into SCFAs, which are produced by breaking down complex carbohydrates and fibre-rich diets as substrates and are dependent on host nutrients. (Tang et al., 2019). SCFAs are responsible for promoting mucus production, thereby improving the intestinal barrier function and inhibiting the transport of lipopolysaccharide, which can lead to endotoxemia (Sandri et al., 2017; Witkowski et al., 2020). A deficiency of SCFAs leads to diseases such as hypertension, diabetes, and obesity, which are risk factors for AF (Zhang et al., 2021). Probiotics as food supplements are being investigated in the treatment of cardiovascular disease and have been shown to reduce TMAO levels (Qiu et al., 2017). In summary, we believed that the use of OACs in AF patients may reduce the relative abundance of the genus *Ruminococcus torques group* and family *Lachnospiraceae*, and increase the abundance of genus *Megamonas*, thereby reducing inflammation, atherosclerosis, and thrombosis. From the nutrition and host metabolism, increasing dietary fiber and carbohydrates and reducing red meat intake may reduce the risk of thrombosis in patients with AF. However, OACs have a two-sided effect, reducing the risk of embolism while increasing the risk of bleeding in the gastrointestinal tract and other parts of the body.We also observed patients experiencing gastrointestinal adverse effects. This study found that OACs could impair renal function, which was consistent with previous studies showing that the treatment of patients with OACs was often associated with renal insufficiency and the risk of renal impairment with NOACs was lower than that with vitamin K antagonists (VKA) (Yao et al., 2017; Chan et al., 2018). Different OACs have different effects on the coagulation system, thus increasing the risk of bleeding. Both rivaroxaban and dabigatran significantly increased the risk of reporting gastrointestinal disorders concerning apixaban and VKA, especially in older patients and females according to the previous report (Pardo-Cabello et al., 2021). These side effects may be associated with certain potential pathogens. This study found that the richness of genera *Escherichia-Shigella* (belonging to phylum *Proteobacteria)*, *streptococcus*, and *Klebsiella* increased in the AF-OAC group. *Streptococcus* bacteria were involved in developing chronic sinusitis, maternofetal immune activation, and gastrointestinal disorders. *Proteobacteria* have been found to cause inflammation and provide an environment conducive to some invading pathogens (Shin et al., 2015). *Escherichia-Shigella* was associated with diarrhea and shown to be able to impair intestinal barrier function, especially the restoration of tight junction protein ZO-1 (Sun et al., 2019; Zhang et al., 2020), indicating that *Escherichia-Shigella* might be a common pathogen underlying gastrointestinal disorder. *Akkermansia* and *Faecalibacterium* were the most abundant species in the non-bleeding subgroup. These bacteria have been found to metabolize dietary components that promote colonic motility, maintain the intestinal immune system, and have anti-inflammatory properties. Patients with CVD often have decreased levels of *Faecalibacterium*, which are BT-producing species identified as important anti-inflammatory commensal bacteria (Dicks et al., 2018). *Akkermensia* also can restore the host mucus layer and has been suggested to contribute to the reduction of metabolic endotoxemia (Everard et al., 2013). Endotoxemia not only induces an inflammatory response but also an intertwined, tissue-factor driven activation of the coagulation system (Schoergenhofer et al., 2017). These extensive findings will pave the way to translate GM use for clinical intervention.

## Limitations

Although these results are encouraging, some limitations of our study should be noted. First, 16S rRNA gene sequencing-based techniques have limitations. In particular, 16S rRNA gene sequencing analysis provides the taxonomic composition of the microbes in the community but does not provide an analysis of the function of the microbiota in AF. Second, this study was unable to determine the precise signaling mechanisms through which the gut microbiota interacts with OACs, but rather elucidated a phenomenon. Untargeted metabolomics is needed to further explain the mechanisms. In addition, many confounding factors, such as medications other than OACs, diet, and lifestyle, may result in bias in the analysis; therefore, a cause-effect relationship cannot be demonstrated. Third, additional large cohort studies and functional research are needed to further investigate the effect of OACs on intestinal microbiota in patients with AF and determine whether exogenous supplementation with *Faecalibacterium* reduces the risk of post-anticoagulated bleeding events. Finally, the single fecal sample from each patient studied here could not provide a dynamic change in the gut microbiota.

## Conclusion

As the first-line agent for thromboembolic prophylaxis in patients with AF, OACs not only prevent thrombosis but also reduce the inflammatory response, which may ease the progression of AF and decrease the risk of thrombosis. The 16S rRNA gene sequencing revealed bidirectional effects of OACs on the intestinal microbiota of patients with AF, which improved the decrease in the number of probiotics while increasing some bacterial genera associated with digestive tract dysfunction. In addition, some microbiota associated with post-anticoagulation bleeding were identified in this study. Finally, supplementation with exogenous *Faecalibacterium* and *Akkermansia* may be a prophylactic strategy for AF-OAC patients to lower the risk of bleeding.

## Data availability statement

The datasets presented in this study can be found in online repositories. The names of the repository/repositories and accession number(s) can be found below: NCBI, PRJNA806986.

## Ethics statement

The studies involving human participants were reviewed and approved by The ethics committee of The First Affiliated Hospital of Gannan Medical University. The patients/participants provided their written informed consent to participate in this study.

## Author contributions

The authors JX, DX, and CR conceived the study and designed the experiments. CL and SZ collected the clinical data. WL and HC interpreted the data and wrote the paper. YZ, HL, and LZ revised the final draft of the paper. YC and XH analyzed data and composed the dataset for publication. All authors contributed to the article and approved the submitted version.

## Supplementary material

Supplementary Figure 1 Gut microbiota diversity analysis in the three groups. **(A)** Alpha diversity was analyzed by the Shannon index. **(B)** Alpha diversity was analyzed by the Shannon index. **(C)** Beta diversity was demonstrated by PCoA based on weighted UniFrac distances.

Supplementary Figure 2
**(A–C)** Alpha diversity among the three anticoagulated subgroups. **(D)** Beta diversity was demonstrated by PCoA based on weighted UniFrac distances in the three anticoagulated subgroups.

Supplementary Figure 3 Proportion of top 30 dominant phyla in the three anticoagulated subgroups.
