# Supplementary figures and images for: Bidirectional effects of oral anticoagulants on gut microbiota in patients with atrial fibrillation
Source: Front Cell Infect Microbiol. 2023 Mar 24;13:1038472. doi: 10.3389/fcimb.2023.1038472 (PMC10080059; doi:10.3389/fcimb.2023.1038472)

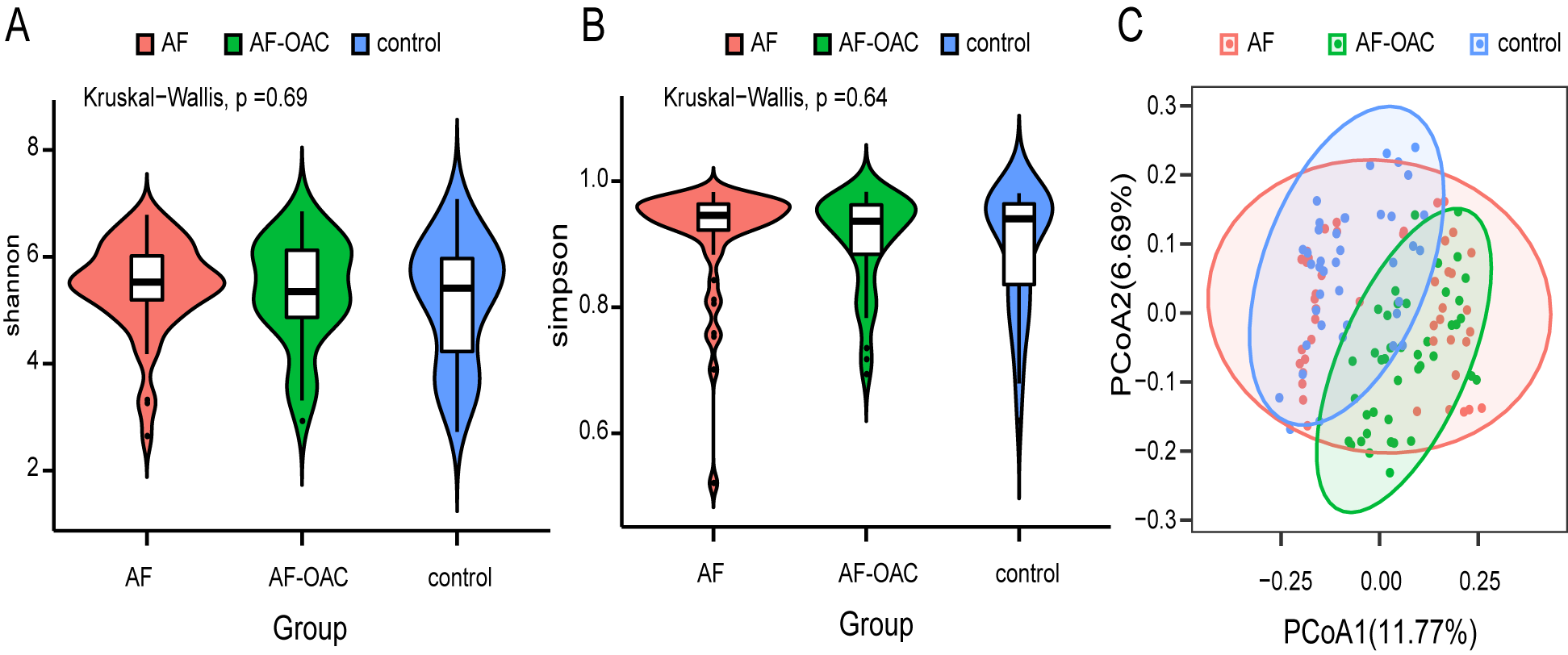

Supplement: Supplementary Figure 1 — Gut microbiota diversity analysis in the three groups. (A) Alpha diversity was analyzed by the Shannon index. (B) Alpha diversity was analyzed by the Shannon index. (C) Beta diversity was demonstrated by PCoA based on weighted UniFrac distances. [file Image_1.tif]

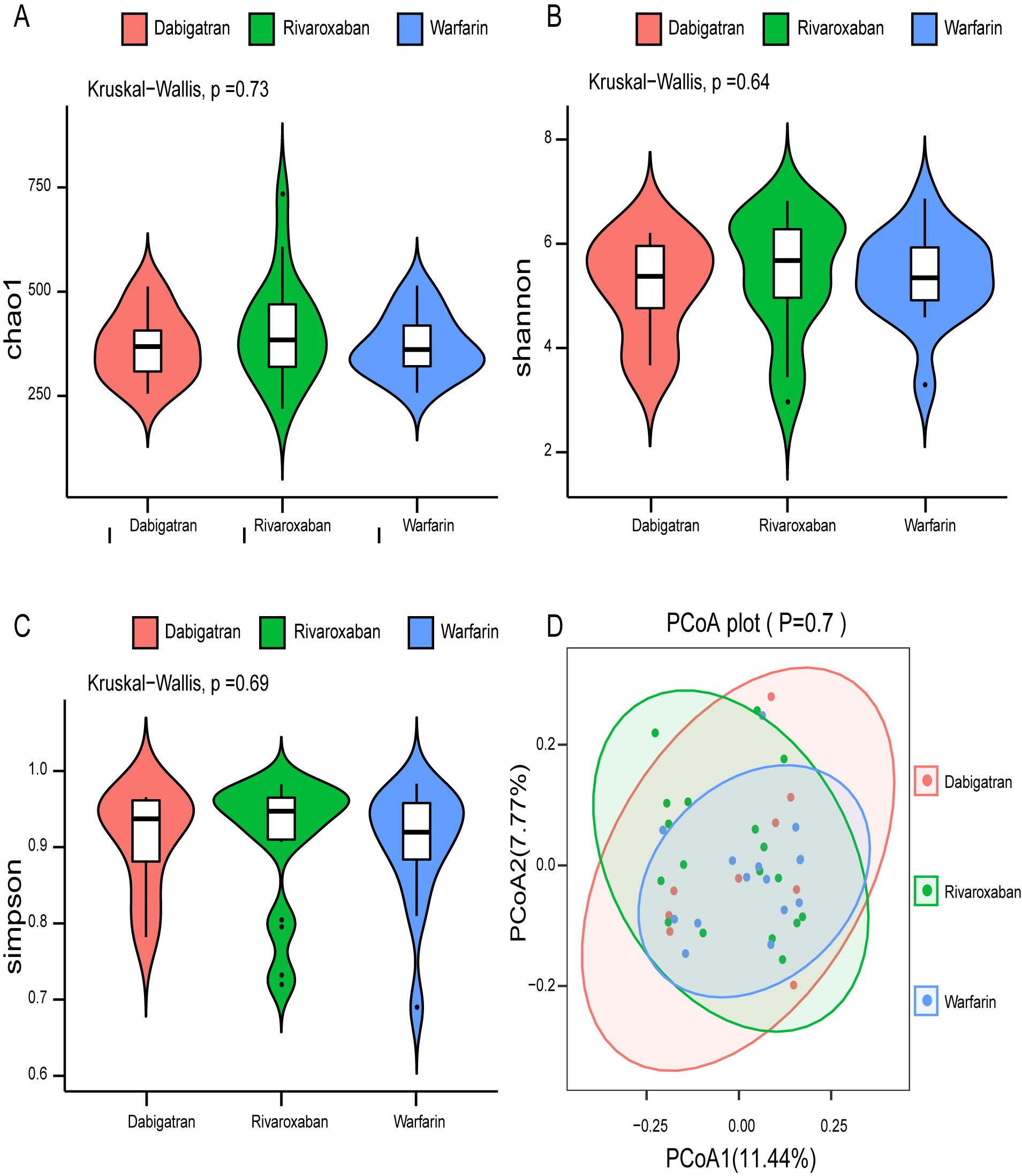

Supplement: Supplementary Figure 2 — (A–C) Alpha diversity among the three anticoagulated subgroups. (D) Beta diversity was demonstrated by PCoA based on weighted UniFrac distances in the three anticoagulated subgroups. [file Image_2.tif]

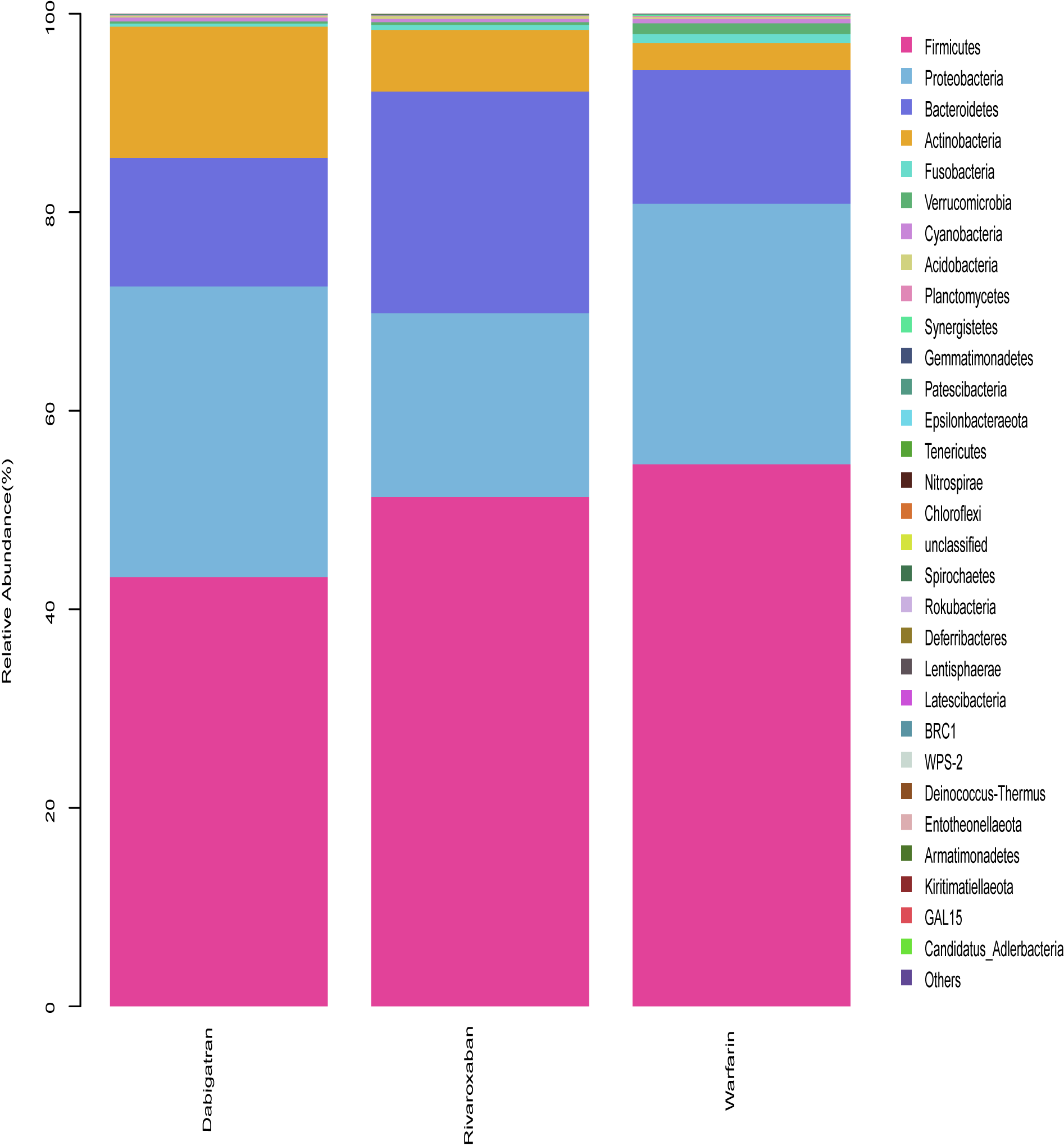

Supplement: Supplementary Figure 3 — Proportion of top 30 dominant phyla in the three anticoagulated subgroups. [file Image_3.tif]
